# Author Correction: The plasmidome associated with Gram-negative bloodstream infections: a large-scale observational study using complete plasmid assemblies

**DOI:** 10.1038/s41467-024-47494-z

**Published:** 2024-04-09

**Authors:** Samuel Lipworth, William Matlock, Liam Shaw, Karina-Doris Vihta, Gillian Rodger, Kevin Chau, Leanne Barker, Sophie George, James Kavanagh, Timothy Davies, Alison Vaughan, Monique Andersson, Katie Jeffery, Sarah Oakley, Marcus Morgan, Susan Hopkins, Timothy Peto, Derrick Crook, A. Sarah Walker, Nicole Stoesser

**Affiliations:** 1 Nuffield Department of Medicine, University of Oxford, Oxford, UK; 2 Oxford University Hospitals NHS Foundation Trust, Oxford, UK; 3 Department of Zoology, University of Oxford, South Parks Road, Oxford, UK; 4 Department of Engineering Science, University of Oxford, Oxford, UK; 5 National Infection Service, United Kingdom Health Security Agency, Colindale, London, UK; 6 NIHR Oxford Biomedical Research Centre, John Radcliffe Hospital, Oxford, UK

**Keywords:** Bacterial infection, Bacterial genes, Clinical microbiology, Antimicrobial resistance

Correction to: *Nature Communications* 10.1038/s41467-024-45761-7, published online 22 February 2024

In this article the author name William Matlock was incorrectly written as Willam Matlock. The original article has been corrected.

