# Supplementary material for: The plasmidome associated with Gram-negative bloodstream infections: A large-scale observational study using complete plasmid assemblies
Source: Nat Commun. 2024 Feb 22;15:1612. doi: 10.1038/s41467-024-45761-7 (PMC10881496; doi:10.1038/s41467-024-45761-7)
Supplement: Supplementary file 1 — Supplementary information [file 41467_2024_45761_MOESM1_ESM.pdf]

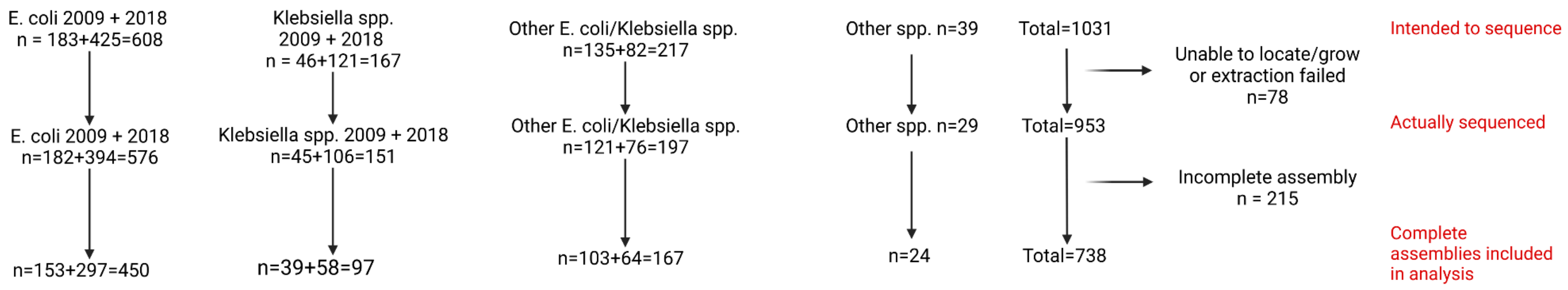

Figure S1: Breakdown of isolates included/excluded in the study by species.

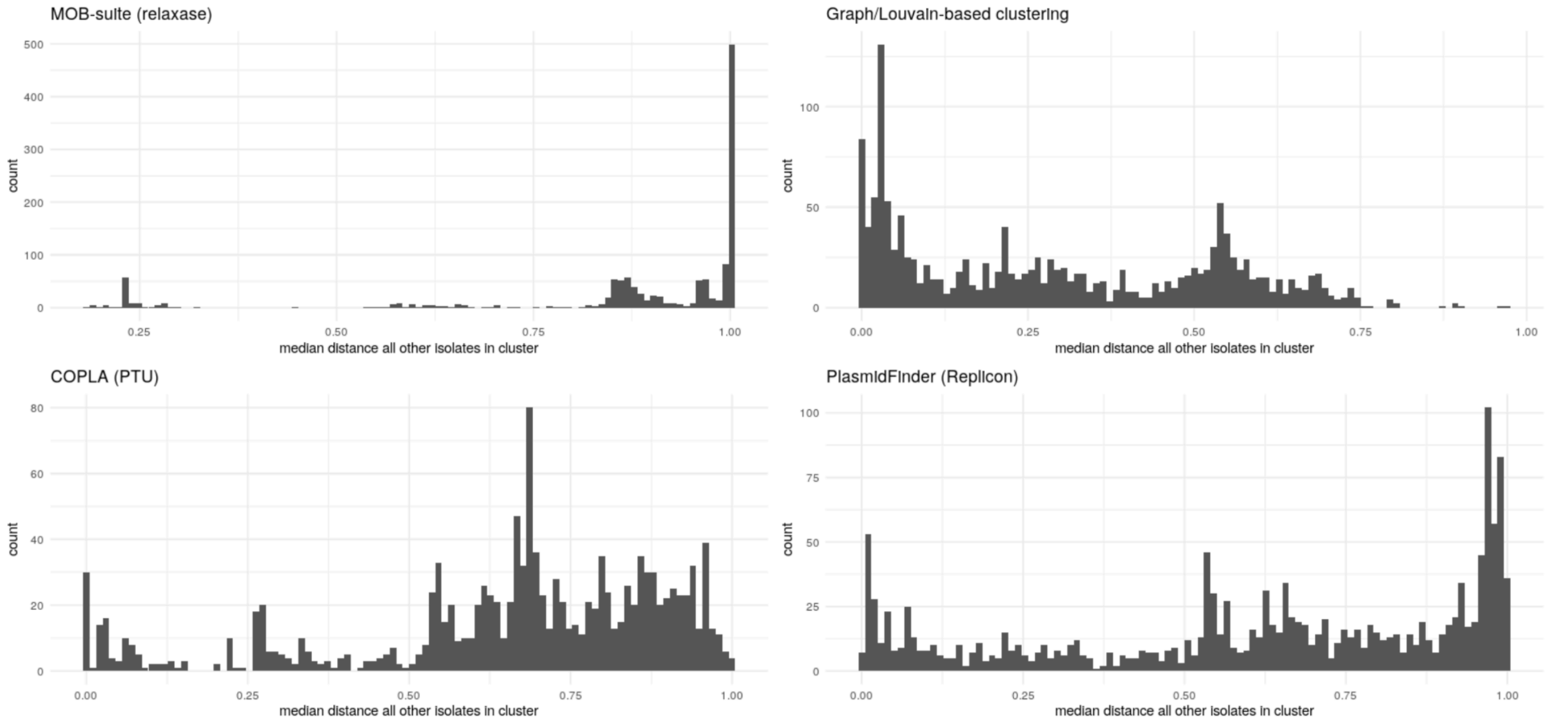

Figure S2: Distribution of distances (defined as in the methods as 1-Jaccard distance from mash output) for plasmids in clusters as defined by the methods shown. Graph/Louvain-based clustering refers to the method used in this manuscript. Source data are provided in the supplementary "Source Data" file.

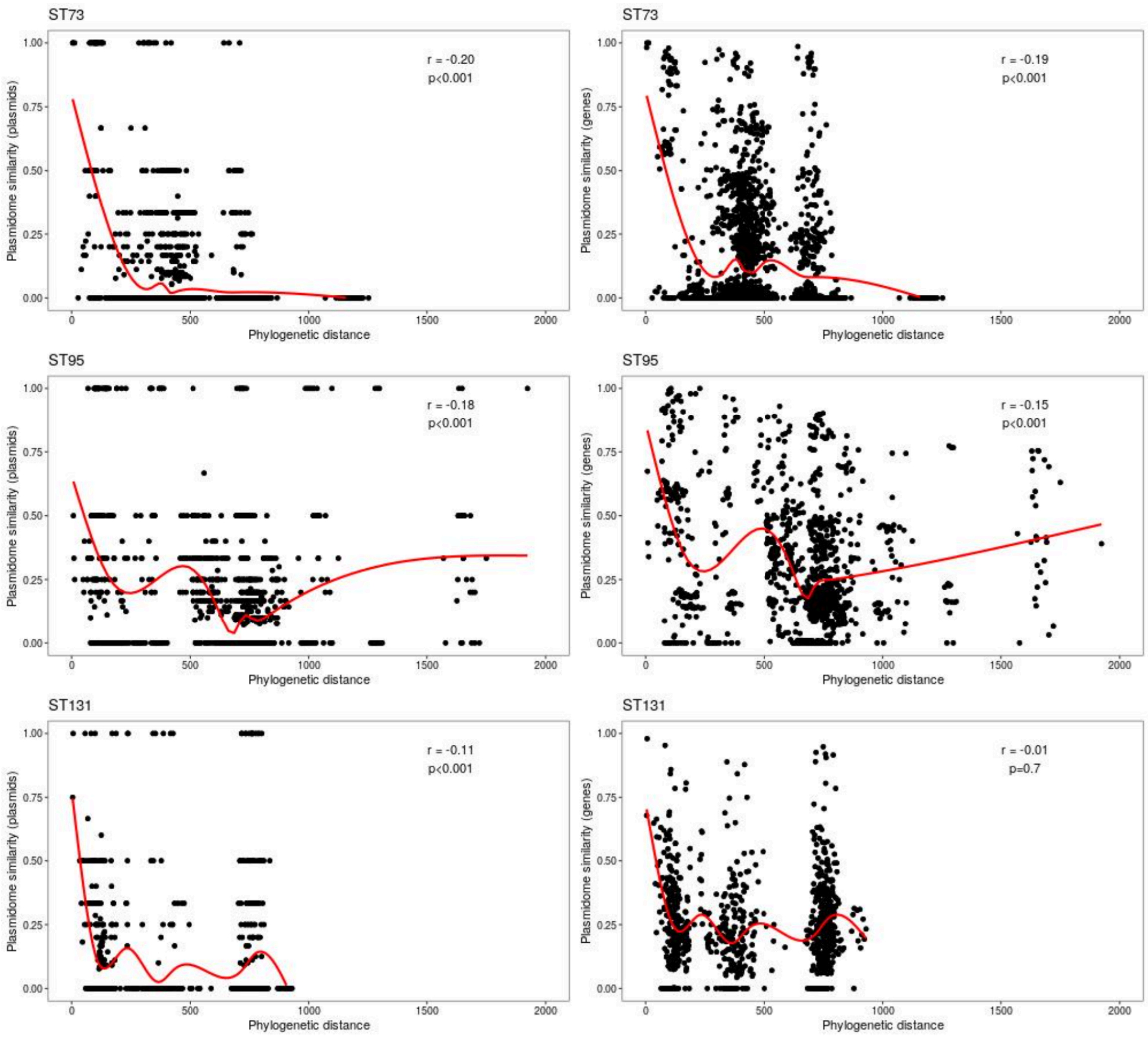

Figure S3: The similarity of the plasmidome is negatively correlated with phylogenetic distance within an ST. Left panel: plasmid similarity in terms of the presence/absence of plasmid clusters. Right panel: plasmid similarity in terms of the presence/absence of plasmid-borne genes. In both cases, the plasmidome similarity between two isolates is 1 minus the Jaccard index (with the Jaccard index involving isolates carrying no plasmids defined as 1). Source data are provided in the supplementary "Source Data" file. Spearman correlations are shown in the plot text with p values shown to 1 decimal place or as <0.001.

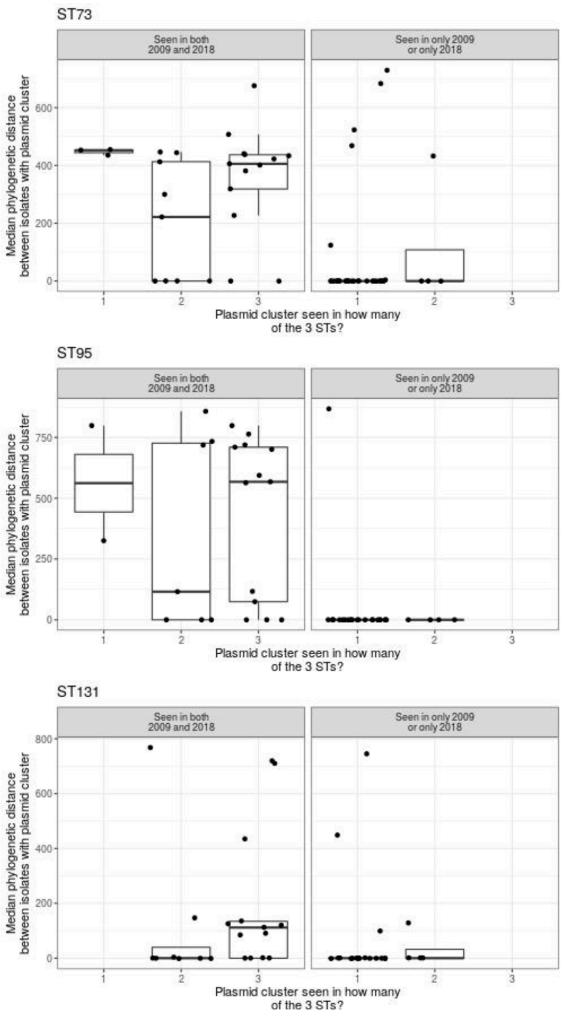

Figure S4: For each of the three selected *E. coli* STs, all the plasmid groups seen in isolates of that ST are shown. Each point shows the median phylogenetic distance between isolates carrying that plasmid cluster, subsetted by the number of STs the plasmid cluster was seen in. Source data are provided in the supplementary "Source Data" file. N=63/49/44 (top/middle/bottom) plasmid clusters. The boxplot hinges show the position of the median and interquartile range with whiskers demonstrating the smallest/largest values no further than 1.5*IQR from the respective hinge.

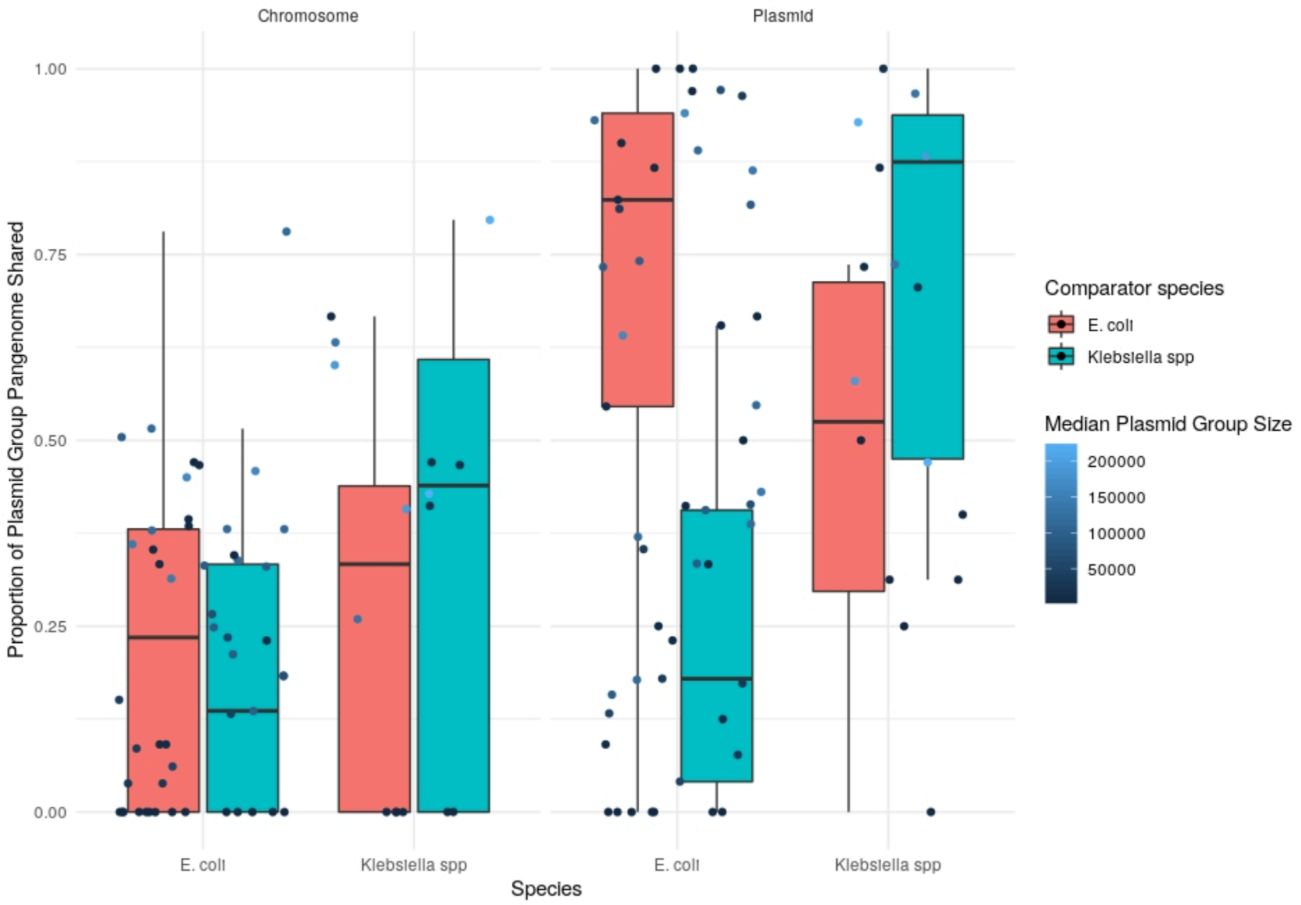

Figure S5: Overlap between plasmid and chromosomal pangenomes. Each dot represents a single plasmid group (n=132, only the largest [n≥10] plasmid groups are shown). The x-axis denotes the predominant host species in which the plasmid group was isolated and the fill colours denote the comparator species. The y-axis position denotes the proportion of genes in the pangenome (as annotated by Prokka/Panaroo) shared between genetic contexts (i.e. plasmid versus chromosome) as indicated. The colour of the dots shows the median plasmid group size. Source data are provided in the supplementary "Source Data" file. The boxplot hinges show the position of the median and interquartile range with whiskers demonstrating the smallest/largest values no further than 1.5*IQR from the respective hinge.

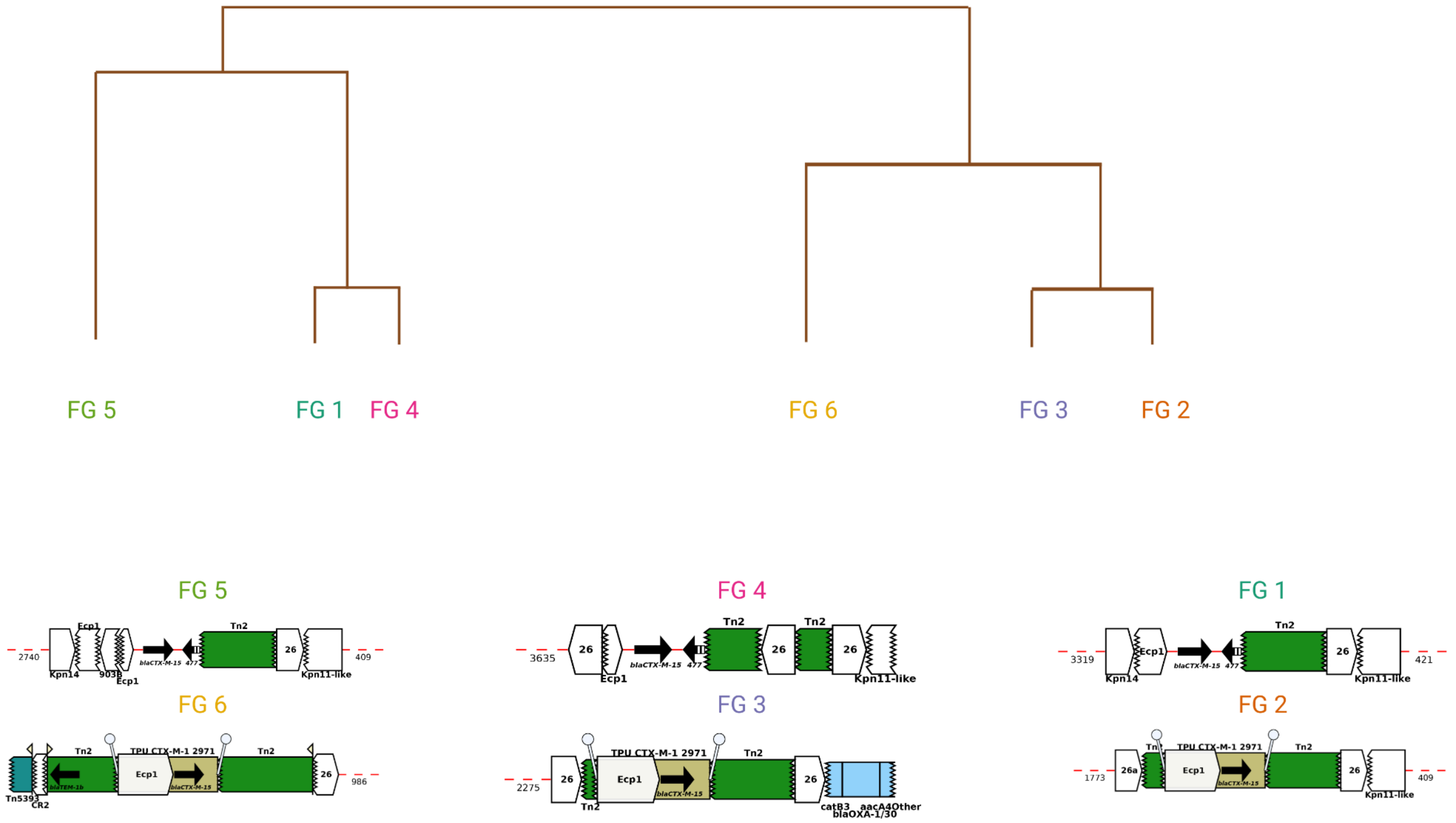

Figure S6: Hierarchical clustering of a weighted graph formed from a distance matrix where the distance between each pair of plasmids was the maximum window length at which Flanker placed them in the same cluster by single linkage. The resulting dendrogram was cut at 0.5 and groups (and nested groups) are shown as identified by RedeR. The lowest levels of these have been renamed sequentially (FG – flank group) and colour coded to match the colours used in Figure 4. The annotations as given by Galileo AMR are shown below for each flank group. Source data are provided in the supplementary "Source Data" file.

Plasmid Group 2

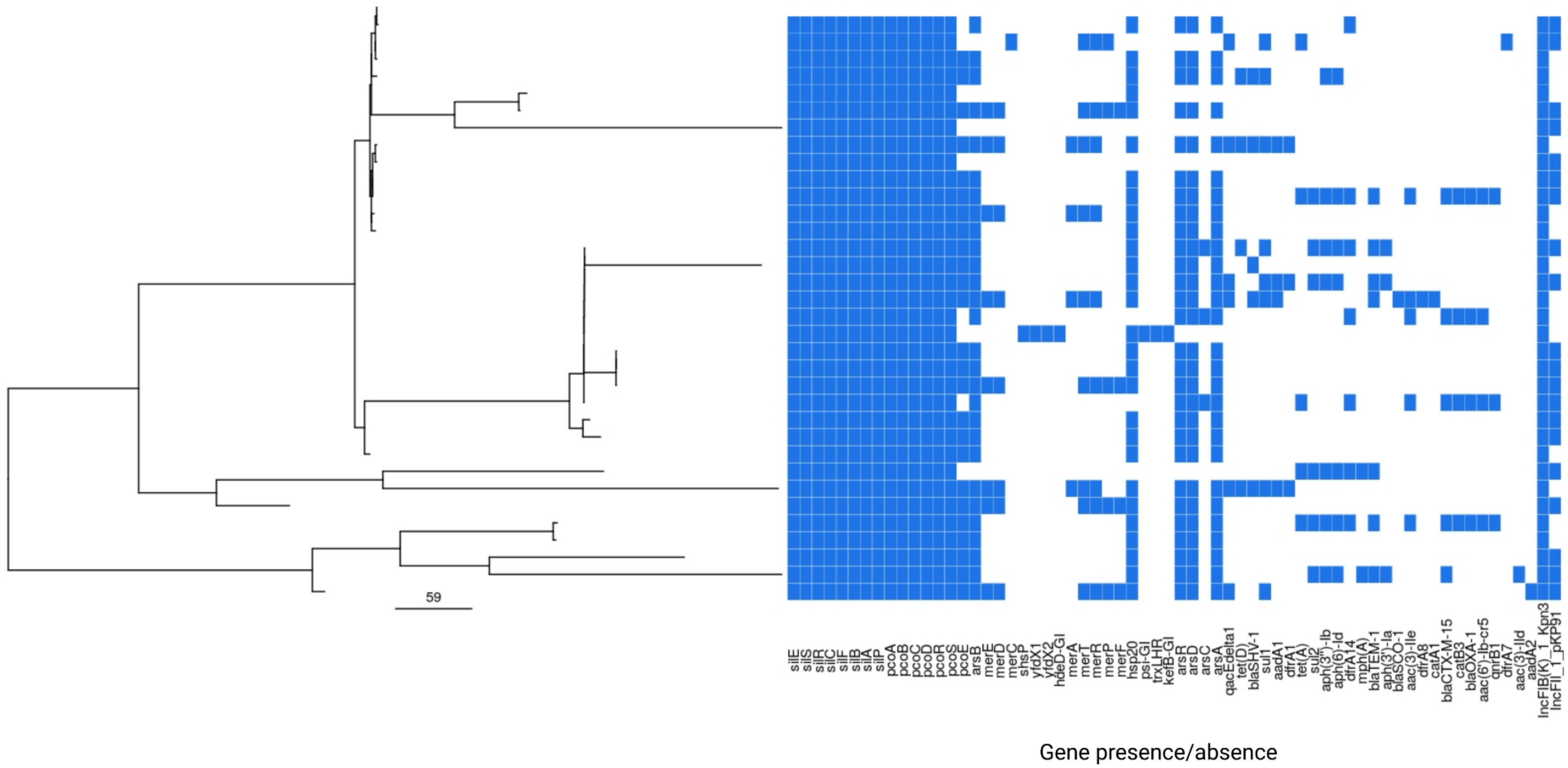

Figure S7: Core genome phylogeny and gene presence/absence heatmap (for genes in the AMRFinder/PlasmidFinder databases). For plasmid group 2 (Figure 2). Source data are provided in the supplementary "Source Data" file.

Plasmid Group 3

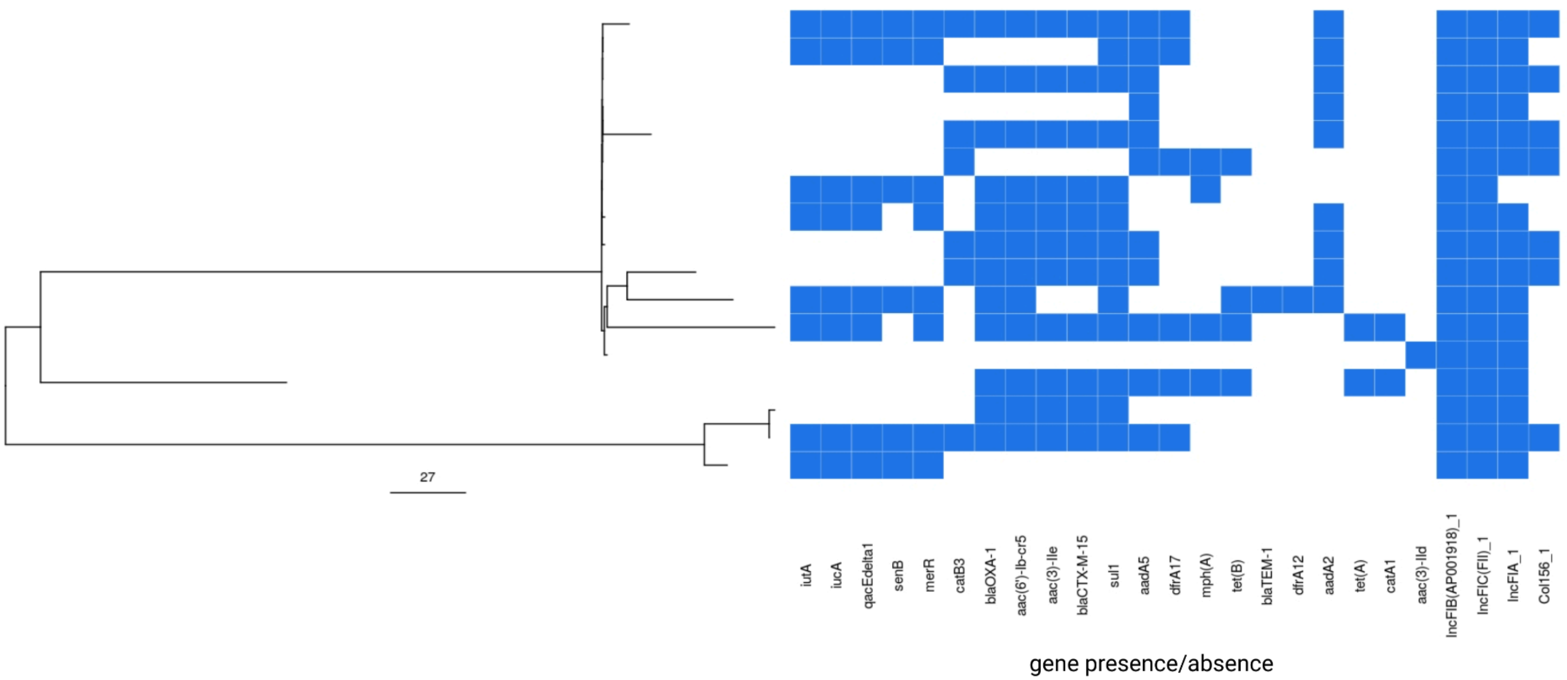

Figure S8: Core genome phylogeny and gene presence/absence heatmap (for genes in the AMRFinder/PlasmidFinder databases). For plasmid group 3 (Figure 2). Source data are provided in the supplementary "Source Data" file.

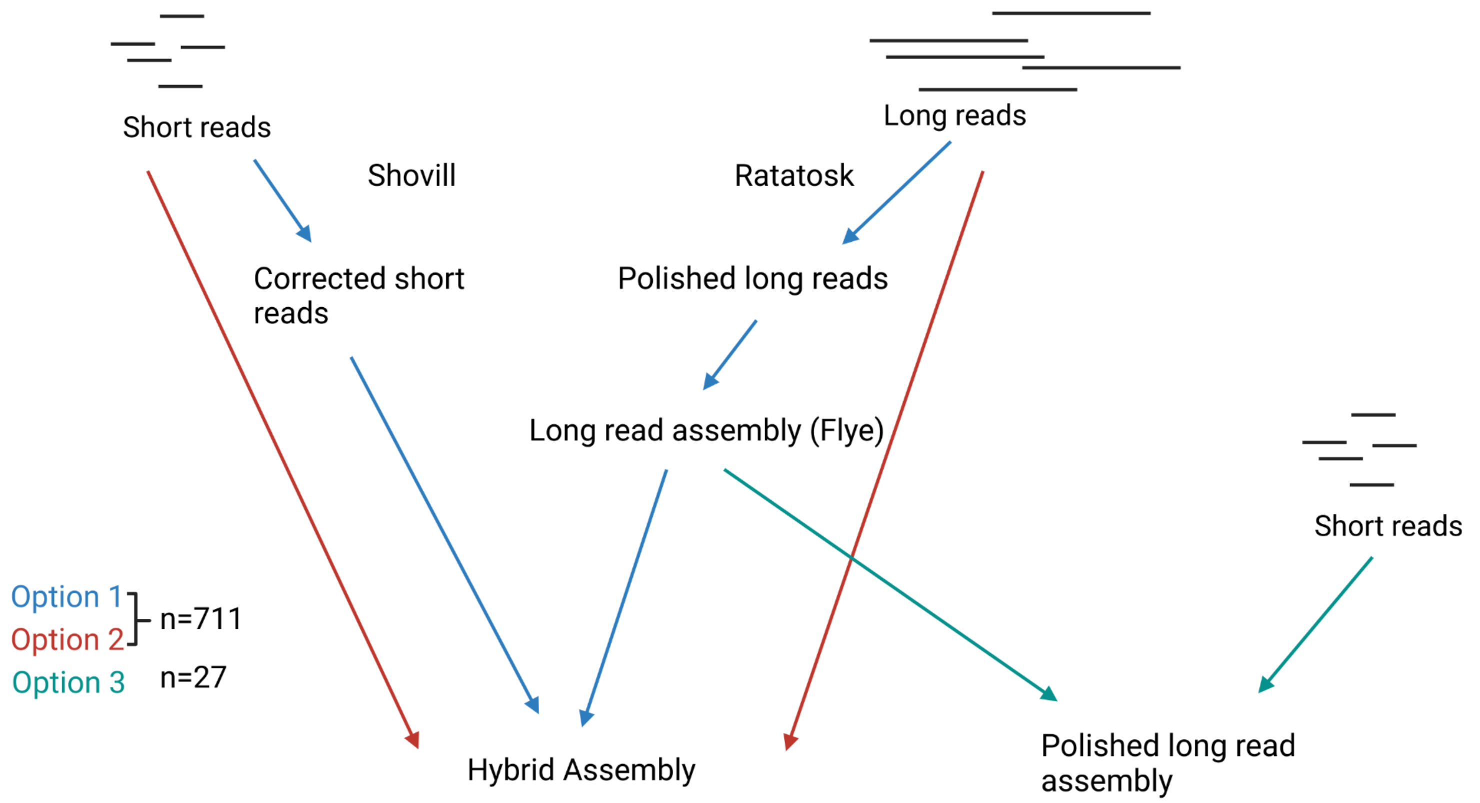

Figure S9: Pictorial representation of assembly pipeline used. Option 1 assemblies were used in preference to option 2, which were preferred to option 3. Hybrid assembly was performed with Unicycler and long read assemblies polished with Pilon.

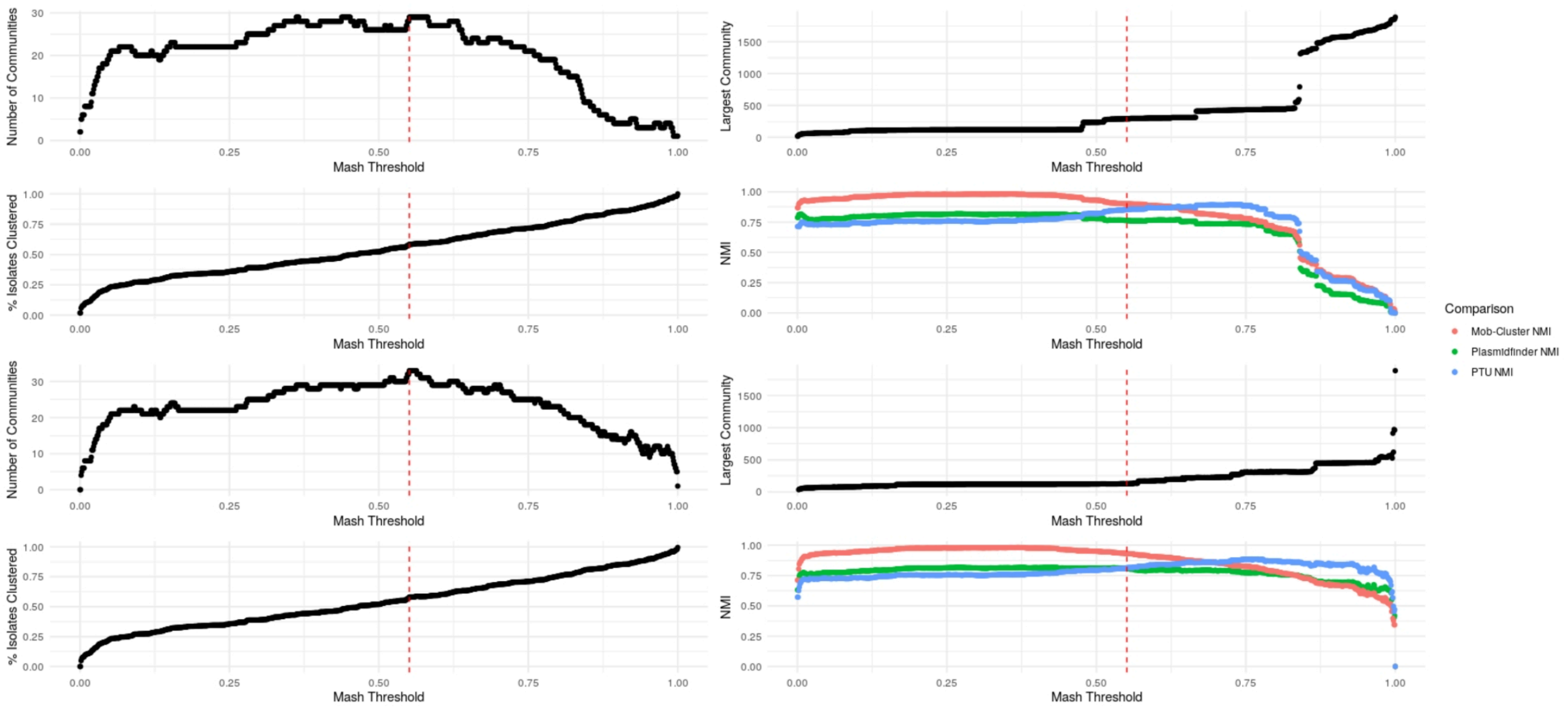

Figure S10: Optimisation of threshold and method to assign plasmids to plasmid groups. The top two rows show the results of a single linkage methodology whereas the bottom two show results obtained using the Louvain clustering algorithm used in our analysis. X-axis shows the Mash distance used to sparsify the group. Number of communities represents the number of plasmid groups n≥10, whereas the largest community represents the largest plasmid group formed at a given threshold. NMI – Normalised mutual information, PTU – plasmid taxonomic unit. The red hashed line shows the Mash distance threshold of 0.551 which was used in the final analysis. Source data are provided in the supplementary "Source Data" file.
