# Supplementary material for: The plasmidome associated with Gram-negative bloodstream infections: A large-scale observational study using complete plasmid assemblies
Source: Nat Commun. 2024 Feb 22;15:1612. doi: 10.1038/s41467-024-45761-7 (PMC10881496; doi:10.1038/s41467-024-45761-7)
Supplement: Supplementary file 3 — Description of Additional Supplementary Files [file 41467_2024_45761_MOESM3_ESM.pdf]

Description of Additional Supplementary Files:

Supplementary Data 1: Assembly metrics/QC.

Supplementary Data 2: Basic properties of plasmids in the dataset.

Supplementary Data 3: Basic epidemiological parameters for isolates included in the study

Supplementary Data 4: Comparison of plasmid clusters produced by the graph/Louvain-based method used in this study compared to other commonly used approaches. All tools/methods were run on the dataset of 1880 plasmids described in this study

Supplementary Data 5a: Counts of plasmids with/without ARGs in the largest PTU groups.

Supplementary Data 5b: Counts of plasmids with/without ARGs in the largest PlasmidFinder groups.

Supplementary Data 6: Plasmid factors associated with plasmid group frequency in the dataset. Poisson models were used and p values are reported to 2 significant figures or as <0.001 where applicable

Supplementary Data 7: Features associated with plasmid groups being unique to Oxford (N=326 groups) or also found in the Acman/global plasmid dataset (N=158 groups) in logistic regression models. There were 5429 plasmid groups only found in the global plasmid dataset. In this analysis plasmids from NCBI were excluded if they were isolated in the UK or if the country of origin was unknown. Statistically significant (p<0.05) variables in the multivariable analysis are highlighted in bold. P values are reported to 2 significant figures or as <0.001 where applicable.

Supplementary Data 8: Genes/gene clusters associated with ARG-carrying plasmids. All values report univariable logistic regression adjusted for plasmid size (categorical large/medium/small) and population structure (10 dimensions of multidimensional scaling of Mash distances between host chromosomes). P values have been adjusted by the Bonferroni method; only significant (p<0.05) results are shown for genes annotated as something other than “hypothetical protein”. Annotations are as given by Prokka/Panaroo. Where Panaroo clustered together multiple prokka annotations, only the first of these is shown. Two plasmids (sizes 1570 and 1308) were excluded from the analysis because they had no coding regions identified. Results are ordered by p value. P-values have been reported to 2 significant figures or as <0.001 as appropriate.
