# Supplementary material for: The plasmidome associated with Gram-negative bloodstream infections: A large-scale observational study using complete plasmid assemblies
Source: Nat Commun. 2024 Feb 22;15:1612. doi: 10.1038/s41467-024-45761-7 (PMC10881496; doi:10.1038/s41467-024-45761-7)
Supplement: Supplementary file 6 — Reporting Summary [file 41467_2024_45761_MOESM6_ESM.pdf]

nature portfolio

Corresponding author(s): Lipworth
Last updated by author(s): Jan 15, 2024

Reporting Summary

Nature Portfolio wishes to improve the reproducibility of the work that we publish. This form provides structure for consistency and transparency in reporting. For further information on Nature Portfolio policies, see our Editorial Policies and the Editorial Policy Checklist.

Statistics

For all statistical analyses, confirm that the following items are present in the figure legend, table legend, main text, or Methods section.

n/a	Confirmed	
☐	☒	The exact sample size (*n*) for each experimental group/condition, given as a discrete number and unit of measurement
☐	☒	A statement on whether measurements were taken from distinct samples or whether the same sample was measured repeatedly
☐	☒	The statistical test(s) used AND whether they are one- or two-sided<br>*Only common tests should be described solely by name; describe more complex techniques in the Methods section.*
☐	☒	A description of all covariates tested
☐	☒	A description of any assumptions or corrections, such as tests of normality and adjustment for multiple comparisons
☐	☒	A full description of the statistical parameters including central tendency (e.g. means) or other basic estimates (e.g. regression coefficient) AND variation (e.g. standard deviation) or associated estimates of uncertainty (e.g. confidence intervals)
☐	☒	For null hypothesis testing, the test statistic (e.g. *F*, *t*, *r*) with confidence intervals, effect sizes, degrees of freedom and *P* value noted<br>*Give P values as exact values whenever suitable.*
☒	☐	For Bayesian analysis, information on the choice of priors and Markov chain Monte Carlo settings
☒	☐	For hierarchical and complex designs, identification of the appropriate level for tests and full reporting of outcomes
☒	☐	Estimates of effect sizes (e.g. Cohen's *d*, Pearson's *r*), indicating how they were calculated

Our web collection on statistics for biologists contains articles on many of the points above.

Software and code

Policy information about availability of computer code

Data collection	No software was used for data collection.
Data analysis	The following open source pieces of software were used for analysis: MLPlasmids v2.1.0, Dashing v1, LinkComm v1.0-14, Guppy v3.1.5, Deepbinner v0.2.0, Unicycler v0.4.9, Flye v2.9-bl768, Ratatosk v0.7.6.3, Shovill v1.1.0, Pilon v1.24, Micropan v2.1, AMRFinder plus v3.10.23, ABRicate v1.0.1, TADB v2.0, Prokka v 1.14.6, MOB-suite v.3.0.0,Panaroo v.1.2.8, Umap v0.2.10, Vegan v2.6-4, Flanker v0.1.5, RedeR v3.18, Mash v2.3, COPLA v1, Igraph v1.6.0, NMI v2.0, R v4.1, Python v3.7.7, Scikit-learn v1.3.2, ggplot v3.4.4, clinker v0.0.28, Cytoscape v3.8.0

For manuscripts utilizing custom algorithms or software that are central to the research but not yet described in published literature, software must be made available to editors and reviewers. We strongly encourage code deposition in a community repository (e.g. GitHub). See the Nature Portfolio guidelines for submitting code & software for further information.

Data

Policy information about availability of data

All manuscripts must include a data availability statement. This statement should provide the following information, where applicable:

- Accession codes, unique identifiers, or web links for publicly available datasets
- A description of any restrictions on data availability
- For clinical datasets or third party data, please ensure that the statement adheres to our policy

Sequencing data for the "Oxfordshire dataset" has been deposited under NCBI project accession PRJNA604975 (https://www.ncbi.nlm.nih.gov/bioproject/PRJNA604975) and at https://doi.org/10.25452/figshare.plus.24573268. Sequencing data for the global dataset (https://doi.org/10.1038/s41467-020-16282-w) is available from the NCBI RefSeq repository ftp://ftp.ncbi.nlm.gov/refseq/release/plasmid. Source data are provided with this paper.

Research involving human participants, their data, or biological material

Policy information about studies with human participants or human data. See also policy information about sex, gender (identity/presentation), and sexual orientation and race, ethnicity and racism.

Reporting on sex and gender	not applicable
Reporting on race, ethnicity, or other socially relevant groupings	not applicable
Population characteristics	not applicable
Recruitment	not applicable
Ethics oversight	not applicable

Note that full information on the approval of the study protocol must also be provided in the manuscript.

Field-specific reporting

Please select the one below that is the best fit for your research. If you are not sure, read the appropriate sections before making your selection.

☒ Life sciences ☐ Behavioural & social sciences ☐ Ecological, evolutionary & environmental sciences

For a reference copy of the document with all sections, see nature.com/documents/nr-reporting-summary-flat.pdf

Life sciences study design

All studies must disclose on these points even when the disclosure is negative.

Sample size	We included all E. coli and Klebsiella spp. BSI isolates in 2009 and 2018 from Oxfordshire UK. A stratified selection of isolates from intervening years were included as described in the manuscript. No formal sample size calculation was performed since the primary intention was to perform a descriptive analysis of isolates consecutively collected at the extremes of time periods for which we were collecting isolates. Whilst the number of isolates included was ultimately constrained by cost and time considerations, we demonstrate in the analysis that the sample encompasses all of the expected diversity of sequence types for E. coli/K pneumoniae that cause invasive human infection.
Data exclusions	Isolates were excluded if the failed to sequence or the assembly was incomplete -see figure S1
Replication	Replication is not applicable since hybrid sequencing produces reference grade assemblies (e.g. see 10.1099/mgen.0.000294 and 10.1099/mgen.0.000132). The very small numbers of SNPs produced on repeated sequencing do not represent real biological signal and would not affect the interpretation of this study.
Randomization	Randomization is not relevant to this study as it is retrospective and observational, there is no intervention.
Blinding	Blinding is not relevant to this study as it is retrospective and observational, there is no intervention.

Reporting for specific materials, systems and methods

We require information from authors about some types of materials, experimental systems and methods used in many studies. Here, indicate whether each material, system or method listed is relevant to your study. If you are not sure if a list item applies to your research, read the appropriate section before selecting a response.

Materials & experimental systems

n/a	Involved in the study
☒	☐ Antibodies
☒	☐ Eukaryotic cell lines
☒	☐ Palaeontology and archaeology
☒	☐ Animals and other organisms
☒	☐ Clinical data
☒	☐ Dual use research of concern
☒	☐ Plants

Methods

n/a	Involved in the study
☒	☐ ChIP-seq
☒	☐ Flow cytometry
☒	☐ MRI-based neuroimaging

Plants

Seed stocks	*Report on the source of all seed stocks or other plant material used. If applicable, state the seed stock centre and catalogue number. If plant specimens were collected from the field, describe the collection location, date and sampling procedures.*
Novel plant genotypes	*Describe the methods by which all novel plant genotypes were produced. This includes those generated by transgenic approaches, gene editing, chemical/radiation-based mutagenesis and hybridization. For transgenic lines, describe the transformation method, the number of independent lines analyzed and the generation upon which experiments were performed. For gene-edited lines, describe the editor used, the endogenous sequence targeted for editing, the targeting guide RNA sequence (if applicable) and how the editor was applied.*
Authentication	*Describe any authentication procedures for each seed stock used or novel genotype generated. Describe any experiments used to assess the effect of a mutation and, where applicable, how potential secondary effects (e.g. second site T-DNA insertions, mosiacism, off-target gene editing) were examined.*
